# Comparative Genomics Analysis of the Populus Epidermal Pattern Factor (EPF) Family Revealed Their Regulatory Effects in *Populus euphratica* Stomatal Development

**DOI:** 10.3390/ijms251810052

**Published:** 2024-09-19

**Authors:** Mingyu Jia, Ying Wang, Hongyan Jin, Jing Li, Tongrui Song, Yongqiang Chen, Yang Yuan, Honghong Hu, Ruting Li, Zhihua Wu, Peipei Jiao

**Affiliations:** 1 Xinjiang Production and Construction Corps Key Laboratory of Protection and Utilization of Biological Resources in Tarim Basin, College of Life Science, Tarim University, Alar 843300, China; jiamingyu0717@163.com (M.J.); 15235060710@163.com (Y.W.); j2601803054@163.com (H.J.); jing926819729@163.com (J.L.); songtr20001209@163.com (T.S.); huhh@mail.hzau.edu.cn (H.H.); 2 Key Laboratory of Crop Genetic Improvement, Hubei Hongshan Laboratory, Huazhong Agricultural University, Wuhan 430070, China; chenyq2017@163.com (Y.C.); yangyuan9109@163.com (Y.Y.); 3 College of Life Sciences, Zhejiang Normal University, Jinhua 321004, China; rutingli@zjnu.edu.cn

**Keywords:** *Populus euphratica*, *EPF* genes, drought stress, stomata, genetic family identification

## Abstract

Drought stress seriously threatens plant growth. The improvement of plant water use efficiency (WUE) and drought tolerance through stomatal regulation is an effective strategy for coping with water shortages. Epidermal patterning factor (EPF)/EPF-like (EPFL) family proteins regulate stomatal formation and development in plants and thus contribute to plant stress adaptation. Here, our analysis revealed the presence of 14 *PeEPF* members in the *Populus euphratica* genome, which exhibited a relatively conserved gene structure with 1–3 introns. Subcellular localisation prediction revealed that 9 *PeEPF* members were distributed in the chloroplasts of *P. euphratica*, and 5 were located extracellularly. Phylogenetic analysis indicated that PeEPFs can be divided into three clades, with genes within the same clade revealing a relatively conserved structure. Furthermore, we observed the evolutionary conservation of *PeEPFs* and *AtEPF/EPFLs* in certain domains, which suggests their conserved function. The analysis of cis-acting elements suggested the possible involvement of *PeEPFs* in plant response to multiple hormones. Transcriptomic analysis revealed considerable changes in the expression level of *PeEPFs* during treatment with polyethylene glycol and abscisic acid. The overexpression of *PeEPF2* resulted in low stomatal density in transgenetic lines. These findings provide a basis for gaining insights into the function of *PeEPFs* in response to abiotic stress.

## 1. Introduction

Water deficit exhibits a broad effect on various plant functions, such as growth, photosynthesis and metabolic pathways. A severe water deficit can result in tissue damage and death [1]. Plants use intricate mechanisms, which also involve the fine regulation of multiple signalling pathways, to respond to drought stress [2,3]. Small secreted peptides (SSPs) refer to a class of peptide molecules with a length of less than 120 amino acids and possess unique functions. As new and important signalling molecules, SSPs not only transmit information from cell to cell over short distances but also contribute to the regulation of plant response to abiotic stress via long-distance information transmission [3]. Plant SSPs exhibit a wide distribution in monocotyledonous and dicotyledonous plants and varying expression levels depending on plant organs. Therefore, these molecules participate not only in the growth and development process but also in the plant response to abiotic adversity stresses [4,5]. The epidermal patterning factor (EPF)/EPF-like (EPFL) family in *Arabidopsis thaliana* encodes a class of cysteine-rich secretory peptides with eleven members. Its precursor proteins have a signal peptide at the N-terminal end and a mature peptide sequence at the C-terminal end, and it is processed by shear to form a mature peptide of about 50 amino acids. It plays an important role in the regulation of stomatal development and stem tip meristem homeostasis [6,7]. Given that the *EPF* gene family function is conserved across several species [8], we hypothesised that some members of the *EPF* family also play an important role in regulating stomatal development in *Populus euphratica* to enhance its stress tolerance performance.

Plants respond to dehydration stress through physiological adjustments, whose regulation depends on the expressions of specific genes involved in the dehydration stress response [9,10]. Studies on plant adversity biology focus on the mechanism of leaf response to drought stress because leaves are essential for photosynthesis and response to environmental changes [11,12,13]. During long-term interaction with the environment, a series of transcription and environmental factors synergistically regulate epidermal cells. This condition eventually leads to their classification into an average of two kidney-shaped guard cells (GCs), which then form stomata [14]. Stomata are an essential innovation in land plants, and their pattern and density are subject to genetic and environmental control [15]. Stomata persist on the epidermis of nearly all terrestrial plant organs above ground. In most dicotyledons, stomata adhere to the ‘one-cell spacing’ rule, where at least one nonstomatal epidermal cell intervenes between two stomata to regulate gas exchange [14]. Furthermore, critical developmental regulatory processes, such as asymmetric cell division, fate transformation, signal transduction and polarity establishment, exhibit a close association with stomatal formation and development [14,16,17,18,19]. Stomata play a crucial role in sensing environmental changes. The opening and closing status and the distribution of stomata serve as key factors in plants’ adaptation to the external environment [20]. In response to environmental changes, plants can regulate carbon dioxide (CO_2_) uptake and water loss through the control of the aperture or density size of stomata. This phenomenon crucially contributes to the regulation of drought resistance, heat tolerance and other adversity stresses. Changes in stomatal traits can improve photosynthesis and water use efficiency (WUE) of plants to a certain extent [21,22,23]. With the assistance of hormones, plants can transiently regulate stomatal aperture to limit water loss under short-term drought stress, such as abscisic acid (ABA) [24]. Conversely, under prolonged drought stress, plants reduce water consumption via the decrease in stomatal density or permanent contraction of the leaf area [25]. Plants can adapt to drought environments by inducing stomatal closure through variations in reactive oxygen species in exosomes, which inhibit transpiration to enhance WUE, and adjusting the density of leaf stomata for acclimation to drought-stressed environments [26,27]. Therefore, the investigation of stomatal formation and its regulatory mechanisms is highly critical for improved water utilisation and coping with drought stress in agricultural production. In various plant species, such as *A*. *thaliana* [28], *Physcomitrella patens* [29], *Horvulgrae* [30], *Oryza sativa* and *Zea mays* [31], multiple members of the *EPF* family have been associated with stomatal development. The *EPF* family genes have been functionally validated in many species. Overexpression of *OsEPF1* and *OsEPF2* genes resulted in decreased stomatal density and significantly increased drought tolerance in *Oryza sativa* [32], whereas overexpression of *OsEPFL9* increased the stomatal density of *Oryza sativa,* thereby decreasing the drought tolerance of the plant [31]. *PdEPF1* and *PdEPF2* genes enhanced the drought tolerance of *Populus tomentosa* by regulating the increase in the stomatal density [33,34], while *Bna.EPF2* enhanced the drought tolerance of *Brassica napus* by regulating stomatal development and stomatal size.

The epidermal patterning factor (EPF)/EPF-like (EPFL) family plays a crucial role in the regulation of stomatal density and distribution via the control of the spacing and segregation of epidermal stomatal development on *A. thaliana* leaf cells [35]. In *A. thaliana*, there are 11 members of the *EPF/EPFL* family, including *AtEPF1* (epidermal patterning factor 1, *At5g62230*), *AtEPF2* (*At5g07180*) [36] and *AtEPFL1*–*AtEPFL9* (*AtSTOMAGEN* and *At4g12970*) [37,38]. These members comprise cysteine-rich peptides, which are primarily secreted extracellularly [36]. The first identified gene (*AtEPF1*) is expressed in late meristemoid mother cells (MMCs), guard mother cells and early GCs. On the other hand, *AtEPF2* is specifically expressed in early stomatal lineage ground cells to prevent cell differentiation towards MMCs [25,28,39]. Furthermore, *AtEPF1* and *AtEPF2* mainly function on the epidermis to inhibit the regulation of stomatal development and distribution at specific times. Meanwhile, *AtEPFL9* and *AtEPFL6* (*AtCHAL* and *At2g30370*) [40] influence stomatal development through intertissue signalling between the epidermal and internal tissues of leaves and stems, respectively [41,42]. *AtEPFL9* is currently the sole positive regulator identified in *A. thaliana*; it belongs to the *AtEPF/EPFL* negative regulatory family, which coordinates stomatal differentiation with a photosynthetic capacity through competitive binding to the same receptor as the negative regulator [37,38]. Heterologous overexpression of *HvEPF1* [30], *OsEPF1*, *OsEPF2* [31] and wheat *TaEPF1B* and *TaEPF2D* [43] in *A. thaliana* resulted in a considerable reduction of stomatal density in *A. thaliana*, which led to an increase in WUE. Studies on *Populus deltoides* [44] and *Brassica napus* [45] have reported similar results, which demonstrated that *EPF* genes can reduce stomatal density and improve WUE. In addition, the overexpression of *TaEPFL1* in *A. thaliana* resulted in shortened filaments and abnormal angiosperm development, which affected stamen development [46].

With the increase in computing power and the rapid expansion of biological data, the use of bioinformatics to solve some biological problems is gradually becoming a mainstream solution. In this article, we mainly applied commonly used bioinformatics analysis software and online websites to identify the *EPF* family members present in the genome of *P. euphratica* and preliminarily predicted the physicochemical properties, chromosomal localisation, conserved structural domains, cis-acting elements and evolutionary relationships with other species of *PeEPFs*. Preliminary predictions of the potential functions of *PeEPFs* were made.

*P*. *euphratica* is utilised as a model organism for various studies due to its remarkable in vitro regeneration capacity, rapid inorganic reproduction and ecological and economic importance throughout the northern hemisphere [47,48]. Given its resilience to drought and salt, *P. euphratica* not only serves as a pioneer species of desert oases but also as a rare relict plant that thrives in extremely arid deserts [49]. Meanwhile, the biological properties of the *EPF* family have been extensively studied in several species, but limited research has been conducted on *Populus*. In this work, we aimed to investigate the specific regulatory functions of *PeEPFs* in the growth mechanism of *P. euphratica* through a comprehensive understanding of the biological characteristics of the *EPF* family. Our findings not only provide new insights into the role of *PeEPFs* in response to abiotic stress but also contribute new gene resources for genetic breeding research involving *P. euphratica*.

## 2. Results

### 2.1. Identification and Prediction of the Physicochemical Properties of PeEPF Family Members

Fourteen *PeEPFs* were identified in the *P. euphratica* genome. Predictive analysis of the physicochemical properties of *PeEPF* family members was performed using the online website ExPASy (https://web.expasy.org/protparam, accessed on 29 November 2023). The PeEPF proteins ranged from 108 amino acids (*PeuTF02G02358.1* and *PeuTF18G01200.1*) to 155 amino acids (*PeuTF07G01021.1*) in length, with molecular weights (Mw) ranging from 11 kDa (*PeuTF18G01200.1*) to 17 kDa (*PeuTF07G01021.1*). The isoelectric point (pI) values of PeEPF proteins varied from 6.98 (*PeuTF03G00338.1*) to 9.96 (*PeuTF11G01022.1*). The instability coefficients, which indicate protein stability in a test tube, ranged from 47.74 to 76.04. This result suggests the considerable variation in the length and physicochemical property of PeEPF proteins, which is possibly related to their diverse functions. A negative average coefficient of hydrophobicity (GRAVY) was observed for all 14 members of the family, which indicates that all the PeEPFs are hydrophilic proteins. Furthermore, subcellular localisation prediction revealed that most of the PeEPFs were located in chloroplasts, and five were detected in the extracellular matrix. Table 1 shows specific details regarding the physicochemical properties.

### 2.2. Analysis of Gene and Protein Structure of PeEPFs

**Figure 1:**
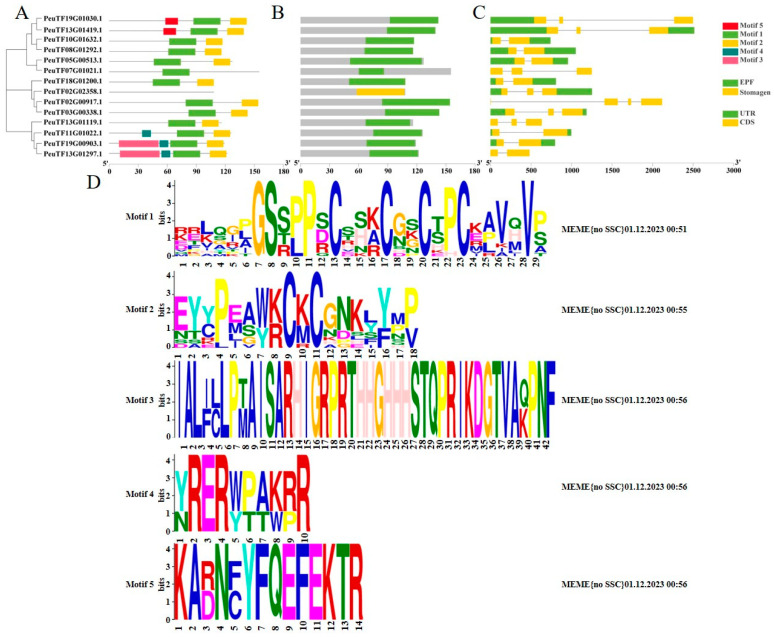
Analysis of gene structure and conserved structural domains of *PeEPFs*. (**A**) Distribution of conserved structural domains of *PeEPFs*. (**B**) Pfam model of *PeEPF*; 13 out of 14 *PeEPF* members have a structural domain model (green), and one gene (*PeuTF02G02358.1*) has a stomagen model such as *AtEPFL9* (yellow). (**C**) Gene structures of *PeEPFs*. (**D**) Conserved motif analysis of *PeEPF* proteins.

The gene structure of each member of the *P. euphratica EPF* gene family, including intron and exon group maps, was analysed based on the positional information of the genes. The results reveal that each member had 2–3 exons (Figure 1C). The conserved protein structural domains of *P. euphratica* were identified via MEME (motif-based sequence analysis tools) analysis. The amino acid sequence of *P. euphratica EPFs* can be divided into five modules (motif 1 to motif 5) (Figure 1D). Analysis of conserved structural domains revealed 2–4 conserved structural domains in the genes of this family in *P. euphratica*. Protein motif module analysis unveiled the presence of motifs 1 and 2 in most *EPFs*, which suggests a high level of conservation for these motifs. Motif 4 was detected in three genes (*PeuTF11G01022.1*, *PeuTF19G00903.1* and *PeuTF13G01297.1*), and motifs 3 and 5 were present in two genes (*PeuTF19G00903.1* and *PeuTF13G01297.1, PeuTF19G01030.1* and *PeuTF13G01419.1*) respectively, which indicates a low level of conservation for these motifs (Figure 1A). Analysis of the Pfam conserved structural domains of the *PeEPF* family members revealed that 13 out of 14 members possess the *EPF* structural domain, and *PeuTF02G02358.1* has the stomagen structural domain, which is consistent with the Pfam model of *AtEPF/AtEPFL* (Figure 1B).

### 2.3. Prediction of Cis-Acting Elements in the Promoter Regions of PeEPFs

**Figure 2:**
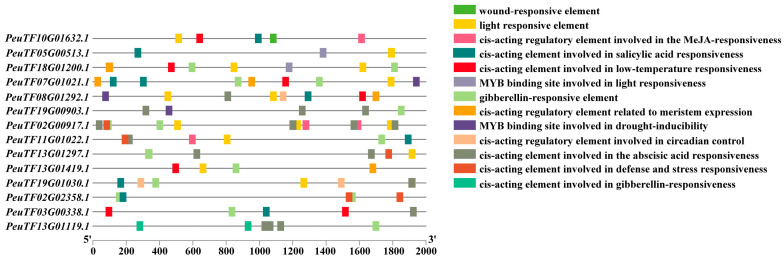
Cis-elements in the promoter region of *PeEPFs*. Colour bars indicate the classification of cis-elements.

To analyse the biological processes involved in *PeEPFs*, we examined the promoter sequence of the 2000 bp region upstream of ATG of *PeEPFs*. The prediction of cis-acting elements revealed their distinct distributions among family members. The elements were primarily categorised into three major groups: hormone-, light- and stress-responsive elements. Stress-responsive cis-acting elements mainly show an association with defence stress, drought stress response and low-temperature response. Phytohormone-responsive elements include salicylic acid response elements (TCA elements), gibberellin response elements (P box and TATC box), methyl jasmonate response elements (CGTCA motif and TGACG motif) and ABA response elements (Figure 2). In this work, hormone response elements were the most abundant, followed by light-response and stress-related elements.

### 2.4. Collinearity Analysis of EPFs in Multispecies

**Figure 3:**
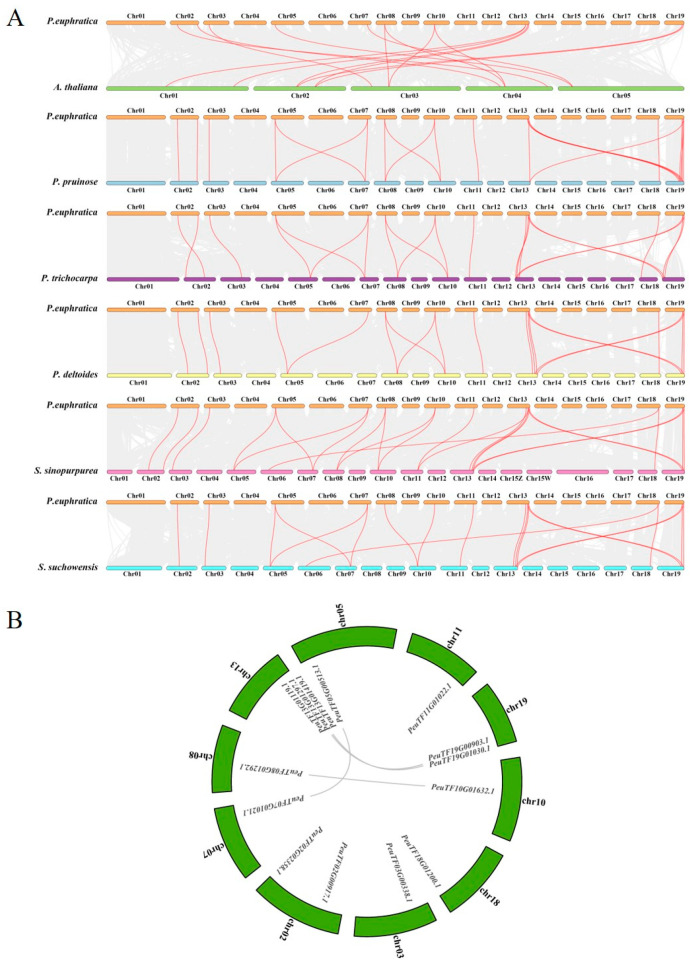
Collinearity analysis of multispecies *EPFs.* (**A**) Collinearity analysis of *EPFs* between *P. euphratica* and five other species (*P. trichocarpa*, *P. deltoides*, *S. sinopurpurea*, *S. suchowensis* and *A. thaliana*). Grey lines in the background indicate the collinear blocks within *P. euphratica* and other plant genomes, and the red lines highlight the collinear *EPF* pairs. (**B**) Intraspecific covariance analysis of *PeEPFs*.

Collinearity analysis falls under two main categories. The first category involves the analysis of genomic collinearity between species to confirm the degree of genomic homology between species. The second focuses on the analysis of homology among various chromosomes within a single species and is used in the distribution assessment of duplication regions or multicopy genes. In this study, the *EPFs* were identified from the genomes of several popular species. A total of 14, 14, 14, 15 and 19 *EPFs* were identified in *P. pruinose*, *P. trichocarpa*, *P. deltoides*, *Salix sinopurpurea* and *Salix suchowensis*, respectively. The potential evolutionary processes of *PeEPFs* were further explored through the comparison of the collinearity of *EPFs* between *P. euphratica* and five other poplar species (*P. pruinose*, *P. trichocarpa*, *P. deltoides*, *Salix sinopurpurea* and *Salix suchowensis*) and *A*. *thaliana*. A total of 22, 21, 12, 21, 24 and 15 *EPF* collinear pairs were identified between *P. euphratica* and *P. pruinose*, *P. trichocarpa*, *P. deltoides*, *Salix sinopurpurea*, *Salix suchowensis* and *A. thaliana*, respectively. This finding suggests that the covariate of *EPF* within poplar species is more conserved compared with that between *P. euphratica* and *A*. *thaliana* (Figure 3A). In addition, eight genes exhibited a collinearity within *P. euphratica* (*PeuTF13G01297.1/PeuTF19G00903.1, PeuTF13G01419.1/PeuTF19G01030.1*, *PeuTF08G01292.1/PeuTF10G01632.1* and *PeuTF05G00513.1/PeuTF07G01021.1*). Each of these four pairs of *P. euphratica* genes may perform the same or similar functions (Figure 3B).

**Figure 4:**
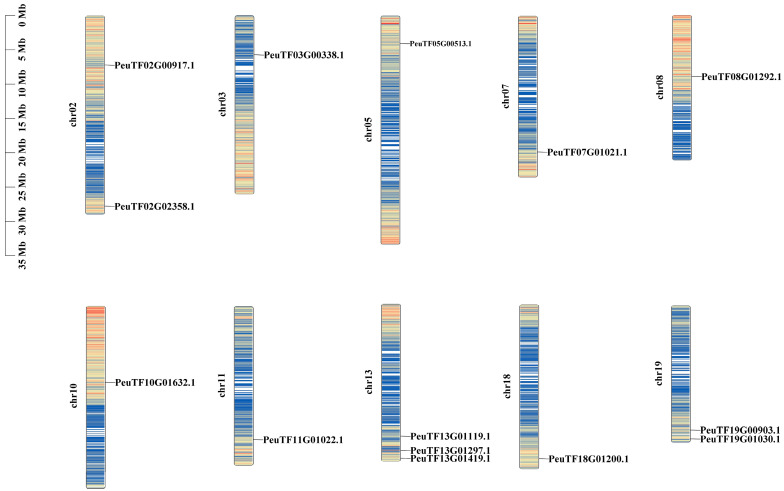
Analysis of the chromosomal localisation of *PeEPFs.* Blue and red bars indicate low and high gene densities on chromosomes, respectively.

Analysis of the chromosomal locations of these 14 *PeEPFs* revealed their distribution on 10 out of the 19 chromosomes of *P. euphratica* (Figure 4). Chromosome 13 contained the largest number of *PeEPFs*, with three *PeEPF* members, followed by chromosomes 2 and 19, with both containing two *PeEPF* members. This uneven distribution on the chromosomes suggests different contributions of each chromosome to the evolution of the *PeEPF* family.

### 2.5. Phylogenetic Tree of PeEPFs

**Figure 5:**
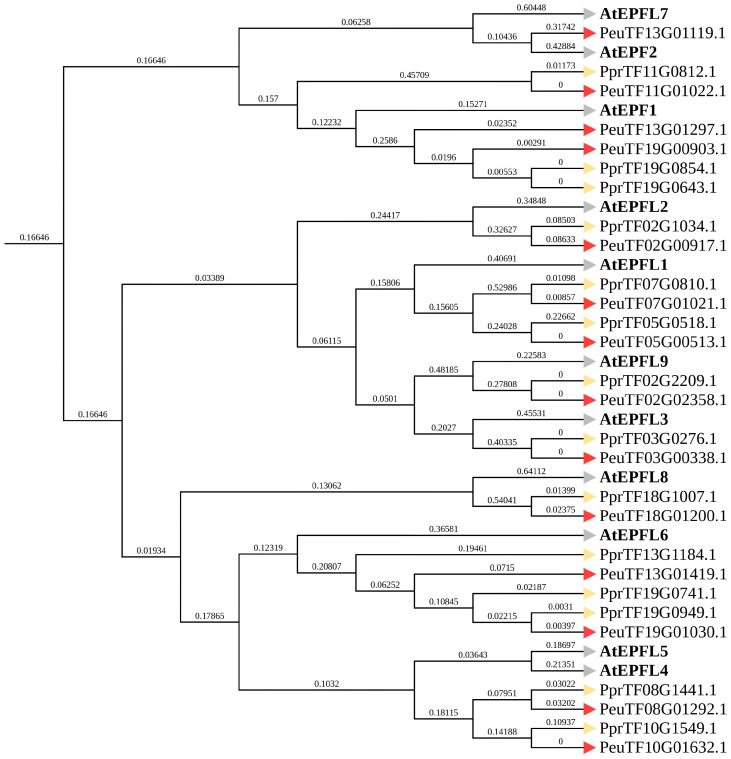
NJ tree of *P. euphratica*, *P. pruinosa* and *A. thaliana*. Phylogenetic trees were constructed using the NJ method using the EPF family protein sequences of the three species. A phylogenetic tree was constructed using MEGA-X (version 11.0.13) software employing the neighbour-joining (NJ) method with 1000 bootstrap replicates. The percentage of replicate trees in which the associated taxa clustered together in the bootstrap test (1000 replicates) is shown next to the branches. The Dayhoff matrix-based method was used to calculate evolutionary distances, which are expressed as the number of amino acid substitutions per site. Ambiguous positions were excluded for each pair of sequences using the pairwise deletion option. TBtools (version 2.119) and the iTOL online website (https://itol.embl.de, accessed on 18 September 2023) were used to visualise the phylogenetic tree. The grey triangles represent *A. thaliana*, the red triangles represent *P. euphratica* and the yellow triangles represent *P. pruinosa*.

To investigate the evolutionary relationships of PeEPF members, we conducted phylogenetic analyses on the protein sequences of 11 AtEPF family members, 14 *Populus pruinose* EPF family members and 14 PeEPF family members. Each EPF family member of *Arabidopsis* had one to three orthologs in *P. euphratica* and *P. pruinosa. P. euphratica* and *P. pruinosa* each had two orthologs on the same branch as AtEPF1 and AtEPFL1 and one ortholog on the same branch as AtEPFL2, AtEPFL3, AtEPFL8 and AtEPFL9. In addition, *P. euphratica* possessed two orthologs, and *P. pruinosa* features three orthologs on the branch of AtEPFL6. The remaining four EPFs were located on the same branch as AtEPFL4 and AtEPFL5. Furthermore, only one ortholog (PeEPF2) was identical to AtEPF2 (Figure 5).

Compared with *Arabidopsis*, *P. euphratica* was found to be more closely evolutionarily related to *P. pruinosa*, with almost every PeEPF corresponding to a PpEPF. In *P. euphratica*, one gene (PeuTF13G01119.1) was directly homologous to AtEPF2. Therefore, in this study, we refer to PeuTF13G01119.1 as PeEPF2.

### 2.6. Transcriptome Sequencing and Data Analysis of PeEPFs

**Figure 6:**
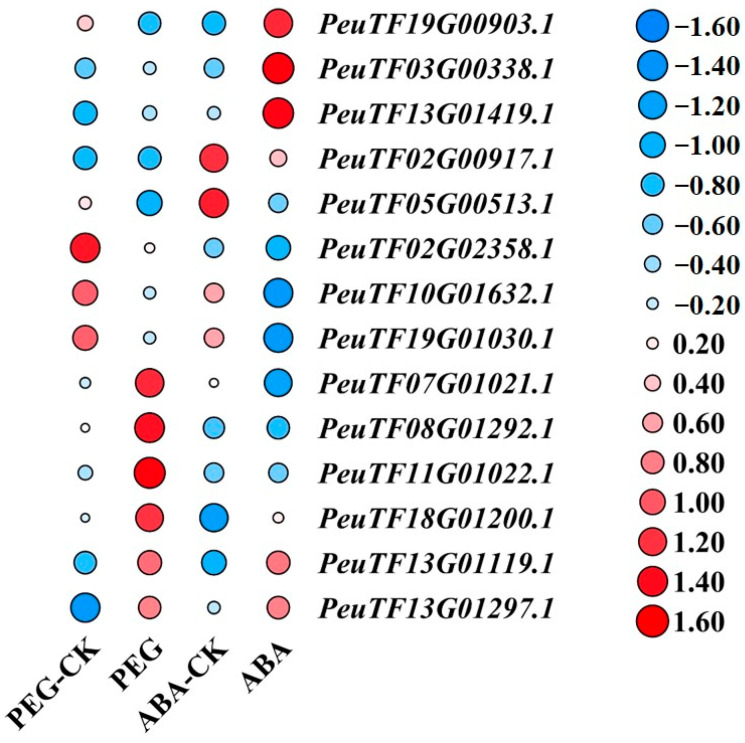
Expression patterns of *PeEPFs* under drought stress and ABA treatment. Heatmap of *PeEPF* expression pattern under drought stress (PEG 6000 treatment) and ABA treatment. The colour scale represents the values of relative gene expression levels; red indicates the high level, and blue denotes the low level of transcript abundance.

To explore the response of *PeEPFs* to drought, we analysed the transcript levels in *P. euphratica* seedlings under drought stress and ABA treatment. The expression data of *PeEPFs* under four conditions (polyethylene glycol (PEG) 6000, ABA treatment and their respective control treatments) were extracted and analysed using TBtools (version 2.119). The results revealed the considerable down-regulation of five genes (*PeuTF02G02358.1*, *PeuTF10G01632.1*, *PeuTF19G01030.1*, *PeuTF02G00917.1* and *PeuTF05G00513.1*) under PEG and ABA treatment. Conversely, four genes (*PeuTF07G01021.1*, *PeuTF11G01022.1*, *PeuTF08G01292.1* and *PeuTF18G01200.1)* were up-regulated under PEG6000 treatment, but they exhibited various changes under ABA treatment. Two genes (*PeuTF13G01119.1* and *PeuTF13G01297.1)* presented increased expressions under PEG and ABA treatments, and three genes (*PeuTF19G00903.1*, *PeuTF03G00338.1* and *PeuTF13G01419.1)* exhibited increased expression under ABA treatment, with less evident changes after PEG treatment (Figure 6). In comparison with other family members, *PeEPF2* revealed regular changes in PEG and ABA treatments and considerable differences in its expression before and after treatments. *PeEPF2* is directly homologous to *AtEPF2* in *A. thaliana* and plays a crucial role in stomatal development. PeEPF2 and AtEPF2 proteins share 70% homology. This finding suggests that *PeEPF2* may be involved in the regulation of stomatal morphogenesis in *P. euphratica*.

### 2.7. Subcellular Localisation of PeEPF2

**Figure 7:**
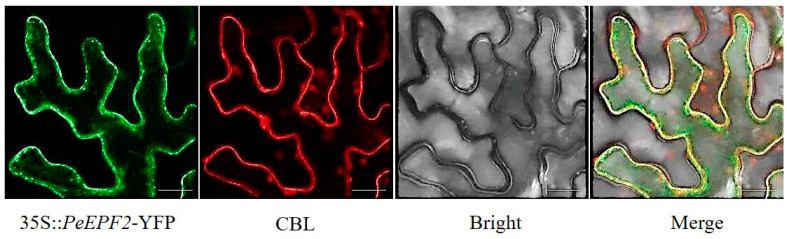
Membrane localisation of 35S::*PeEPF2*-YFP protein in tobacco leaf epidermal cells: PeEPF2 (35S::*PeEPF2*-YFP), membrane localisation signals (CBL) and merged images (35S::*PeEPF2*-YFP/CBL). Scale bar = 25 µm.

The function of molecules is often affected by their localisation within cells. As a signalling molecule, *PeEPF2* may function on or near the cell membrane to reach cell-to-cell contact. To confirm this hypothesis, *Agrobacterium* strains carrying the 35S::*PeEPF2*-YFP construct were introduced into tobacco (*Nicotiana benthamiana*) for the investigation of the subcellular distribution of the PeEPF2 protein in plant cells and observation of its localisation via laser scanning confocal microscopy. The fluorescence signals of the 35S::*PeEPF2*-YFP coincided with the signals of CBL membrane localisation (Figure 7). This finding suggests that *PeEPF2* is mainly expressed on the cell membrane, although the corresponding gene expression usually refers to the transport of the gene-encoded protein to the cell membrane and its biological function there. In *A*. *thaliana*, the AtEPF2 protein acts as a signalling molecule; it transmits signals and influences cell differentiation and proliferation via the interaction with receptors on the cell surface. Therefore, we speculate that the expression of the *PeEPF2* gene on the cell membrane also functions as a signalling molecule.

### 2.8. Effect of PeEPF2 Overexpression on the Regulation of Stomatal Density in Transgenic A. thaliana

**Figure 8:**
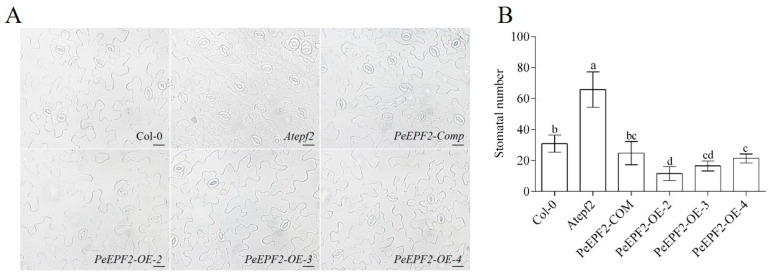
Stomatal numbers of rosette leaves counted on 3-week-old *A. thaliana*: *Atepf2,* Col-0*, PeEPF2-COM, PeEPF2-OE-2, PeEPF2-OE-3* and *PeEPF2-OE-4.* (**A**) Imaging of stomatal number in *Arabidopsis* wild-type *Col-0*, loss-of-function mutant *epf2*, *epf2* complemented with *PeEPF2* lines and *PeEPF2* overexpression lines OE-2, OE-3 and OE-4. Scale bar = 100 µm. (**B**) Statistical analyses of stomatal number in Col-0, *epf2*, *epf2* complemented and *PeEPF2* overexpression with *PeEPF2* plants. Values are means + standard error (each with at least 20 leaves per experiment). One-way analysis of variance was used for statistical analysis; significant differences are indicated by different letters.

The mutation of *AtEPF2* caused an increase in stomatal density, whereas its overexpression resulted in a reduction [50]. To explore the involvement of *PeEPF2* in stomatal development and the similarity of its function to *AtEFP2*, we expressed *PeEPF2* CDS driven by the 35S promoter in *Atepf2* mutant plants, in which the stomatal density was increased [51]. We observed that the expression of *PeEPF2* dramatically reduced the stomatal density of *epf2*. Statistical analyses revealed the lower level of stomatal density of these three randomly selected independent lines than the Columbia type (Col-0) (Figure 8). The findings suggest that *PeEPF2* has a conserved function of *AtEPF2* for the negative regulation of stomatal development.

## 3. Discussion

### 3.1. Identification of 14 EPF Family Members in P. euphratica

*P. euphratica* is a widespread perennial tree in the northwest desert region, and the challenge associated with its irrigation has made drought a primary concern for its productivity and survival in forest ecosystems. Therefore, the accelerated dissection of drought-resistant mechanisms of forest trees and improvement of their adaptive capacity to drought are urgently needed for arid land use, environmentally sustainable development and increased economic efficiency.

Previous studies have demonstrated the importance of peptides as crucial mediators of intercellular interactions in animals. Studies on plant peptides gradually emerged and led to the identification of an increasing number of plant peptide hormones. Plant peptide signals contribute to the regulation of various aspects of plant growth and development, especially in plant cellular communication within plants. The *EPFs* represent a class of small-Mw, cysteine-rich peptide hormones. The first protein in the family, *EPF1*, was published in 2007 for its function in the regulation of stomatal development. Subsequently, reports on *EPFs* and their involvement in stomatal development saw a sudden surge. *EPF1* and *EPF2* act as negative regulators of stomatal development and exert their effects via the common receptors *ER* and *TMM* [52]. On the other hand, *EPFL9* serves as a positive regulator of stomatal development and currently serves as the sole signalling protein identified to have such an effect [37,38,53].

At present, studies on *PeEPFs* are lacking. This study identified 14 *EPF* members in each of *P. euphratica*, *P. pruinosa*, *P. trichocarpa* and *P. deltoides*. Preliminary prediction of its conservativeness and gene function via bioinformatics analysis and its gene structure conservativeness provided theoretical support for subsequent functional verification.

### 3.2. EPF Structure Implies a Conservative Function in Drought Tolerance in P. euphratica

Biologists have continued to pursue the increase in photosynthesis in food crops via the increase in the number of stomata. As a result, active efforts in breeding research have been exerted to boost stomatal density and thereby improve light efficiency, water utilisation and crop yield. Similarly, the reduction of stomatal density to enhance drought tolerance in plants was explored. Previous studies have yielded positive findings regarding the role of *SDD1* in the regulation of plant physiological traits via stomatal density [54]. In addition, since their discovery as peptide hormones, EPFL9 and EPF2 proteins have shown a promising potential for the improvement of plant physiological traits [50].

A recent study on the modification of stomatal density with *EPFL9* and *EPF2* in *A. thaliana* demonstrated their positive effects on the plant’s physiological traits. The effect was considered significant given that the increase in stomatal density in leaves of *EPFL9* overexpressing plants improved the photosynthetic rate by 30% [55]. This phenomenon is not limited to *A. thaliana*; it has also been shown to be effective in *P. deltoides*. A study revealed a 28% reduction in stomatal density of poplar trees overexpressing *PdEPF1*, which led to a nearly 30% decrease in transpiration without affecting CO_2_ uptake. Ultimately, this condition resulted in a dramatic improvement in the drought resistance [33]. Overexpression of *PeABF3* enhanced drought tolerance in *Populus tomentosa* by directly regulating *ADF5* to promote ABA-induced stomatal closure [56]. Overexpression of *PdEPFL6* in 84K poplar reduced poplar stomatal density and enhanced plant drought tolerance, whereas overexpression of *PdEPFL9* promoted plant stomatal production and negatively regulated plant drought tolerance [57]. In our current study, the expression level changes in *PeEPFs* upon PEG and ABA treatments indicate that the members of this family are regulated to respond to drought to ensure plant growth and development under such conditions. Therefore, the gene *PeEPF2* in this study is also promising for expression in *Populus 84K* or other poplar species for its drought tolerance effect. Further experiments are needed.

The *EPF* family has been extensively studied in several species, including the prototypic species *A*. *thaliana*, *Oryza sativa* [58] and *Malus pumila*. A number of genes in this family, particularly *EPF1, EPF2, EPFL6* and *EPFL9,* perform crucial roles in stomatal development and distribution. Phylogenetic analyses involving the *EPF* families of *A. thaliana* and *P. euphratica* revealed the clustering of 14 members of the *EPF* family in *P. euphratica*, and they were analysed alongside 11 members of the *EPF* family in *A. thaliana*. However, only *PeuTF13G01119.1* exhibited a direct homology to *AtEPF2* in the evolutionary tree. Its phenotype revealed an increased stomatal density in *A. thaliana Atepf2* mutants. Similarly, changes in the expression of their *EPF2 orthologs* have been found to affect the stomatal density in other plants, such as *Populus* [59], *Brassica napus* [45], *Hordeum vulgare* and *pumila* [58]. We hypothesised that alterations in the expression of *PeuTF13G01119.1*, the sole ortholog to *AtEPF2* in *P. euphratica*, will likewise affect the stomatal density of *P. euphratica* in a specific manner.

In this article, a preliminary evolutionary analysis of 14 genes in the *PeEPFs* was carried out, and a phylogenetic tree of the *EPFs* was constructed using bioinformatics methods to resolve the evolutionary relationships of the *EPFs*. Through the comparison of protein sequences and the observation of stomatal distribution phenotypes in combination with those in the studied species, it is concluded that the *EPFs* are more conserved in higher plants. At present, we have only carried out preliminary functional validation of the *EPF2* in *P. euphratica*, and the results demonstrate that *PeEPF2* negatively regulates stomatal development in *A. thaliana*, and its function is very close to that of *EPF2* in *A. thaliana*, *Oryza sativa*, *Malus pumila* [31,45,60], etc. Other genes of the *EPF* family, such as *EPF1*, *EPFL6* and *EPFL9*, have been reported in species such as *A. thaliana*, *Oryza sativa* and *Brassica napus*, and they also function very conservatively. *EPF2* is very conserved in *A. thaliana*, *Oryza sativa* and *Malus pumila*. *EPF1* [25] and *EPFL6* [61] negatively regulate stomatal development, and *EPFL9* [37] positively regulates stomatal development. Therefore, we speculate that the genes with high homology to EPF1, EPFL6 and EPFL9 in *PeEPFs* may also play important roles in regulating stomata, which needs to be verified by further experiments.

### 3.3. PeEPF2 Participates in the Regulation of Stomatal Density in Transgenic A. thaliana

*AtEPF2* is involved in stomatal density regulation, and to confirm the possible conservative function of *PeEPF2* in stomatal density regulation, we determined the stomatal numbers of *PeEPF2*-overexpressing plants. The stomatal number in the *PeEPF2*-overexpressing plants was significantly lower than that in the wild type; meanwhile, the stomatal number of the loss-of-function mutant *Atepf2* (SALK_102777) was evidently higher than that of the wild-type Col-0, as reported previously [36]. The number of stomata shows a close relationship to a series of physiological responses, such as leaf temperature and WUE. Thus, *PeEPF2* in *P. euphratica* may affect drought tolerance by influencing the stomatal density of plants, and further experiments are required to verify its function.

## 4. Materials and Methods

### 4.1. Genetic Identification, Multiple Sequence Alignment and Phylogenetic Analysis

PeEPFs were analysed based on the genomic data of *P. euphratica* [62]. The EPFs from four other Salicaceae species were identified using the genome data on *P. pruinose* (National Center for Biotechnology Information (NCBI), with BioProject accession number PRJNA863418), *P. deltoides* (WV94_445) [63], *P. trichocarpa* (V3.1) [47], S*alix sinopurpurea* [64] and *Salix suchowensis* [65].

All genomic data were initially compared with eleven *AtEPF* protein sequences obtained from NCBI using a BLASTp search with an e-value of 1.0 × 10^−10^. In addition, HMMER (https://www.ebi.ac.uk/Tools/hmmer/search/phmmer, accessed on 26 November 2023)was used to identify potential EPF proteins. Genes containing complete CD length-specific *EPF* conserved structural domains were then identified through further screening using the Pfam batch sequence (PF17181; PF16851) search (http://pfam.xfam.org, accessed on 26 November 2023) and NCBI batch CD search (https://www.ncbi.nlm.nih.gov/cdd, accessed on 26 November 2023). Each candidate *EPF* was further confirmed using SMART (http://smart.embl-heidelberg.de, accessed on 26 November 2023) and CDD (https://www.ncbi.nlm.nih.gov/cdd, accessed on 26 November 2023). The pI of the *PeEPF* protein and theoretical Mw were predicted using ExPASy (https://www.expasy.org, accessed on 29 November 2023). Subcellular localisation of the *EPF* gene family members in *P. euphratica* was predicted using the WOLF PSORT online website (https://www.genscript.com, accessed on 29 November 2023).

### 4.2. Analysis of Gene Structure and Conserved Structural Domains of PeEPFs

Conserved motifs were analysed using the web software MEME (http://meme-suite.org, accessed on 1 December 2023). The optimal widths ranged from 10 to 150, with the number of motifs set to 8 and the rest as defaults. Subsequently, the gene structures and motifs were plotted using the mapping tool TBtools (version 2.119), followed by analysis of the results.

### 4.3. Analysis of Cis-Acting Elements of PeEPFs

To analyse the promoter sequences in the *PeEPF* family and predict their functions, we entered 2000 bp sequences upstream of the start codon of *PeEPFs* into the PlantCare website (https://bioinformatics.psb.ugent.be/webtools/plantcare/html, accessed on 13 December 2023) to predict cis-elements in the promoters of each gene. Subsequently, TBtools (version 2.119) software was used to visualise these predictions.

### 4.4. Multispecies Collinearity and Chromosomal Localisation of PeEPFs

BLASTP alignment was performed to identify the orthologous pairs of *P. euphratica* and six other species (*P. pruinose*, *P. trichocarpa*, *P. deltoides*, *Salix sinopurpurea*, *Salix suchowensis* and *A. thaliana*) [66,67]. Subsequently, the blocks between *P. euphratica* and these six other species were screened using TBtools (version 2.119) software and visualised accordingly. Mapping of the chromosomal distribution of *EPFs* and their physical locations in *P. euphratica* was achieved using the information from the *P. euphratica* genome files and annotation files through the TBtools (version 2.119) software.

TBtools (version 2.119) software was used to extract chromosome length information (Fasta Stats), PeEPFs gene ID and position information (GFF3 gene position parse/Text Block Extract and Filter) and gene density information (Gene Density Profile) from the *P. euphratica* genome file. Then, TBtools/Gene Location Visualise was used to visualise the chromosome positions, and TBtools/Gene Location Visualise was used to visualise the chromosome locations.

### 4.5. Phylogenetic Tree Analysis of PeEPFs

The domain coordinates within the *EPF* protein sequence of *P. euphratica* and *A. thaliana* were retrieved using the SMART website (http://smart.embl-heidelberg.de, accessed on 26 December 2023). Extraction of sequences of the *EPF* domain and their merging into a new sequence were performed using its coordinates. Subsequently, the merged protein sequence was used in the construction of the phylogenetic tree of the two species. The merged protein sequences of *EPF*s from *P. euphratica* and *A. thaliana* were aligned using the MUSCLE method of MEGA-X (version 11.0.13) with default settings. After the amino acid sequence alignment, gap trimming was conducted under the Site Coverage Cutoff parameter of 0.95 utilising the Multiple Alignment Trimming tools in the TBtools (version 2.119) software. The evolutionary history was inferred via the neighbour-joining (NJ) method. The bootstrap consensus tree, which was derived from 1000 replicates, was used to represent the evolutionary history. The percentage of replicate trees containing the clustered as-associated taxa in the bootstrap test (1000 replicates) is indicated next to the branches. The Dayhoff matrix-based method was used to calculate evolutionary distances, which are presented as the number of amino acid substitutions per site. All ambiguous positions were removed for each sequence pair using the pairwise deletion option. The phylogenetic tree was displayed using the ITOL online website (https://itol.embl.de, accessed on 18 September 2023) and TBtools (version 2.119).

### 4.6. Transcriptome Sequencing and Data Analysis of PeEPFs

Using the sequencing data obtained from the transcriptome sequencing of *P. euphratica* seedling samples treated with 15% PEG 6000, 100 µmol/L ABA and control conditions in the previous stage of the project and the quantitative expression analysis of genes in each sample [68], Frasergen Bioinformatics Co., Ltd. (Wuhan, China) performed RNA extraction, cDNA library construction, RNA-seq and primary data analysis. After the library was qualified, a DNA nanoball was prepared and loaded onto the sequencing chip, and the MGI high-throughput sequencer was used for sequencing. Off-machine data were processed using the SOAPnuke (version 2.1.0) software [69] to filter raw reads and obtain high-quality clean reads. We referred to transcriptome analysis methods from another article [70]. Hisat2 (version 2.1.0) software [71] was used to compare the collected high-quality Illumina clean reads with the reference genome of *P. euphratica*. The Stringtie (version 1.3.4d) software [72] was used to perform quantitative expression analysis on the genes of each sample. Gene expression levels were quantified as fragments per kilobase per million, which refers to the number of fragments per thousand bases compared with the exon of the reference genome per million reading. R package DESeq2 (version 1.30.1) was used to identify differentially expressed genes (DEGs). The genes with |log_2_ Fold Change| > 1 and *p*-value < 0.05 in a comparison were considered DEGs. The expressions of *PeEPFs* were extracted and visually analysed using TBtools (version 2.119).

### 4.7. Subcellular Localisation of PeEPF2

The constructed 35S::*PeEPF2*-YFP vector was transformed into Agrobacterium tumefaciens GV3101. The strain harbouring the target plasmid (CBL-mcherry) was reconstituted in LB medium supplemented with appropriate antibiotics for overnight cultivation. The bacterial solution obtained in the second step was inoculated into a fresh Luria–Bertani (LB) medium along with the simultaneous addition of acetosyringone and agitated until the bacteria reached an optical density (OD600) of 1.0–1.2. The supernatant was discarded via centrifugation, and the bacteria were resuspended in an infection fluid (0.01 M morpholinoethanesulphonic acid (MES) (pH = 5.6), 0.01 M MgCl_2_·6H_2_O and 50 µM acetosyringone) until the OD reached approximately 1.0. The suspension was left undisturbed for 3 h in a dark environment. The target bacteria 35S::*PeEPF2*-YFP were combined with CBL-mcherry in equal proportions and inoculated in about four-week-old tobacco leaves using a syringe. The treated plants were kept in the dark for 12 h and incubated under normal conditions for 36 h. The underlying epidermis of tobacco was revealed in a dark environment and examined using a laser scanning confocal microscope (TS100, Nikon, Tokyo, Japan). The microscope was excited using a 514 nm laser, and emitted signals were detected within a range of 524–574 nm.

### 4.8. Effect of PeEPF2 Overexpression on Stomatal Number in A. thaliana

The wild-type *A*. *thaliana* seeds used in this experiment were of Col-0. The *Arabidopsis* mutant *epf2* was identified as *Atepf2-1* (SALK_102777) [51]. The *Escherichia coli* competent strain was *E. coli*-Top10, and the *Agrobacterium* strain was GV3101. pgreenII 0179 (35S NOS)-YFP2 was used as the overexpression vector. For a detailed transgenic method of *PeEPF2* in heterologous Arabidopsis, refer to another report [73].

The fifth and sixth rosette leaves of a five-week-old seedling of Col-0, *Atepf2-1*, *PeEPF2-Comp* and *PeEPF2* overexpression lines were obtained. The middle part was cut off, the lower epidermis was pasted onto a transparent adhesive tape facing downwards, and the lower epidermis was pressed gently with a finger to ensure the tight attachment of the lower epidermis to the tape. The upper epidermis and leaf pulp cells were gently scraped off with a sharp blade in one direction and then gently brushed off the remaining leaf pulp cells with a soft-bristled brush moistened with water. Photographs were obtained under a light microscope at 20 times magnification. Six leaves of three plants were collected from each material, with two fields of view for each leaf, and the number of stomata in 12 fields of view were counted using Image J software (version 1.47).

## Figures and Tables

**Table 1:** Characteristics of *PeEPFs*.

Gene ID	Number of Amino Acids	Molecular Weight	Theoretical pI	Instability Index	Aliphatic Index	Grand Average of Hydropathicity (GRAVY)	Prediction of Subcellular Localization
PeuTF19G00903.1	119	13,128.32	9.11	61.06	73.03	−0.124	chlo
PeuTF02G00917.1	154	16,947.5	9.39	51.15	80.45	−0.208	extr
PeuTF02G02358.1	108	12,168.91	7.59	70.48	75.93	−0.278	extr
PeuTF18G01200.1	108	11,850.8	9.41	51.61	65	−0.229	chlo
PeuTF19G01030.1	142	16,263.8	9.06	71.07	48.03	−0.288	chlo
PeuTF05G00513.1	127	14,030.07	8.14	64.9	63.07	−0.262	chlo
PeuTF13G01419.1	139	15,769.57	9.39	50.85	60.29	−0.122	extr
PeuTF13G01297.1	122	13,476.85	9.32	47.74	78.44	−0.025	chlo
PeuTF07G01021.1	155	17,660.55	9.11	48.44	62.97	−0.251	extr
PeuTF13G01119.1	116	12,811.99	8.63	37.3	66.38	−0.088	chlo
PeuTF03G00338.1	143	14,799.81	6.98	53.63	72.38	−0.108	chlo
PeuTF10G01632.1	117	12,978.02	9.58	76.04	66.67	−0.399	chlo
PeuTF08G01292.1	116	12,594.75	9.91	60.47	72.33	−0.123	chlo
PeuTF11G01022.1	126	13,987.56	9.96	50.63	82.78	−0.048	extr

## Data Availability

No large datasets were created in this study.
